# Investigation of Iron Metabolism in Mice Expressing a Mutant Menke’s Copper Transporting ATPase (Atp7a) Protein with Diminished Activity (Brindled; Mo*^Br^* ^/*y*^)

**DOI:** 10.1371/journal.pone.0066010

**Published:** 2013-06-11

**Authors:** Sukru Gulec, James F. Collins

**Affiliations:** Food Science and Human Nutrition Department, University of Florida, Gainesville, Florida, United States of America; Queen Mary University of London, United Kingdom

## Abstract

During iron deficiency, perturbations in copper homeostasis have frequently been documented. Previous studies in iron-deprived rats demonstrated that enterocyte and hepatic copper levels increase and a copper transporter (the Menkes Copper ATPase; Atp7a) is induced in the duodenal epithelium in parallel to iron transport-related genes (e.g. Dmt1, Dcytb, Fpn1). Moreover, two ferroxidase proteins involved in iron homeostasis, hephaestin expressed in enterocytes and ceruloplasmin, produced and secreted into blood by the liver, are copper-dependent enzymes. We thus aimed to test the hypothesis that Atp7a function is important for the copper-related compensatory response of the intestinal epithelium to iron deficiency. Accordingly, iron homeostasis was studied for the first time in mice expressing a mutant Atp7a protein with minimal activity (Brindled [Mo*^Br^* ^/*y*^]). Mutant mice were rescued by perinatal copper injections, and, after a 7–8 week recovery period, were deprived of dietary iron for 3 weeks (along with WT littermates). Adult Mo*^Br^* ^/*y*^ mice displayed copper-deficiency anemia but had normal iron status; in contrast, iron-deprived Mo*^Br^* ^/*y*^ mice were iron deficient and more severely anemic with partial amelioration of the copper-deficient phenotype. Intestinal iron absorption in both genotypes (WT and Mo*^Br^* ^/*y*^) increased ∼3-fold when mice consumed a low-iron diet and ∼6-fold when mice were concurrently bled. WT mice exhibited no alterations in copper homeostasis in response to iron deprivation or phlebotomy. Conversely, upregulation of iron absorption was associated with increased enterocyte and liver copper levels and serum ferroxidase (ceruloplasmin) activity in Mo*^Br^* ^/*y*^ mice, typifying the response to iron deprivation in many mammalian species. We thus speculate that a copper threshold exists that is necessary to allow appropriate regulate of iron absorption. In summary, Mo*^Br^* ^/*y*^ mice were able to adequately regulate iron absorption, but unlike in WT mice, concurrent increases in enterocyte and liver copper levels and serum ferroxidase activity may have contributed to maintenance of iron homeostasis.

## Introduction

Iron is an essential trace mineral that serves as a cofactor for enzymes which mediate diverse biochemical reactions including oxygen delivery, energy metabolism and immunity. Overall body iron homeostasis is primarily controlled by absorption of dietary iron in the upper small bowel, as no regulated excretory pathways exist in mammals. Properly managing body, tissue and cellular iron levels is critical as free iron can generate oxygen free-radicals, causing damage to biological molecules (e.g. DNA) and membranes. A detailed understanding of molecular mechanisms mediating *trans*-epithelial iron transport is thus critical to develop therapeutic approaches to modulate iron absorption in humans with pathologies that perturb iron homeostasis (e.g. iron overload- and iron deficiency-related disorders).

Previous investigations have noted that copper influences iron homeostasis [1]–[3]. During iron deficiency, in many mammalian species, body copper levels increase [4], [5], including in the intestinal mucosa [6], the liver [7] and in serum [8]. Consistent with this, we noted induction of a copper exporter, the Menkes copper-transporting ATPase (Atp7a), in duodenal tissue extracted from iron-deficient rats [9]. Atp7a pumps copper into the *trans*-Golgi network of intestinal epithelial cells (IECs) for cuproenzyme synthesis and upon copper excess, traffics to the basolateral membrane to mediate copper efflux [10]. Upregulation of Atp7a is thus consistent with documented alterations in intestinal and body copper levels during iron deficiency.

Atp7a is necessary for copper absorption, as exemplified in patients with Menkes disease [11] and in mice harboring *Atp7a* mutations [12], which present with copper loading in enterocytes (and other tissues) and severe systemic copper deficiency. Given its’ necessity for assimilation of dietary copper, Atp7a may mediate increases in body copper levels during iron deficiency. Moreover, Atp7a (and copper) may be a key player in the compensatory response of the intestinal epithelium to increase body iron acquisition during states of deficiency. The role of copper in iron homeostasis is best exemplified by the multi-copper ferroxidases (FOX), hephaestin (Heph) and ceruloplasmin (Cp). Heph is a membrane-bound FOX expressed in enterocytes, and is important for iron efflux [13], while Cp is a liver-derived, circulating FOX that mediates iron release from stores in liver and spleen [14]. During iron deficiency, Cp expression and activity increases, possibly due to increased metallation of the *apo*-enzyme in copper-loaded hepatocytes [15]. Heph is also induced during iron deficiency [16], but whether copper accumulation in enterocytes directly influences Heph expression or activity is not known.

The current investigation was undertaken to test the hypothesis that Atp7a function is important to maintain body iron homeostasis during states of deficiency. Atp7a could directly influence enterocyte or liver copper levels, potentially increasing expression or activity of the multi-copper FOXs. Our approach was to utilize Brindled (Mo*^Br^* ^/*y*^) mice, which have a 6 base-pair deletion in the *Atp7a* gene [17], resulting in a protein with significantly reduced functional activity [18]. To assess the role of Atp7a specifically in iron homeostasis, neonatal mutant mice were rescued by copper injection, allowing them to live beyond 14-days-of-age when they would normally die from severe copper deficiency. Once mutant mice recovered, they along with wild-type (WT) littermates were deprived of dietary iron, and then body iron and copper homeostasis was studied. Results showed that rescued Brindled mice suffered from copper-deficiency anemia, in which hemoglobin, hematocrit and body copper is low, but iron levels are normal. When mutant mice were deprived of iron, they had a similar ability as WT mice to upregulate intestinal iron absorption. However, unlike the situation in WT mice, where no alterations in copper levels were noted, increases in enterocyte and liver copper content and serum ferroxidase activity occurred concomitantly with enhanced iron absorption in mutant mice.

## Methods

### Ethics Statement

All studies on experimental animals were approved by the University of Florida IACUC and they were performed according to the guidelines of the America Association for Laboratory Animal Sciences.

### Animal Procedures

Mottled brindled (Mo*^Br^* ^/*y*^) mice on a CBA/C3H background were provided by Dr. Julian Mercer (Deakin University, Australia), and a breeding colony was established at the University of Florida. The X-linked *Atp7a* gene has a six bp mutation in Mo*^Br^* ^/*y*^ mice resulting in a two amino acid deletion in the 4^th^ *trans*-membrane segment [17], abolishing activity of the phosphatase domain. Brindled mice have been used widely as a model of Menke’s disease in humans [19], [20]. Brindled (Mo*^Br^* ^/+^) females were bred to wild type males (Mo^+/y^). Screening for the Brindled mutation was done by examination of the coat color; hemizygous males displayed a markedly hypopigmented coat and curly whiskers whereas heterozygous Brindled females had a mottled coat. Newborn Mo*^Br^* ^/*y*^ mice were rescued by 2 injections of CuCl_2_ (50**µg in 20 µL of 0.9% NaCl) into the scruff of the neck at 7- and 9-days-of-age. After Cu injections, mice were carefully monitored and experiments were performed when they were ∼3 months old. Mice were fed semi-purified AIN-93G rodent diets for 21 days prior to sacrifice (Dyets Inc., Bethlehem, PA). The control diet contained 198 ppm iron and the low-iron diet contained 3 ppm iron, with the diets being otherwise identical. These are diet formulations that we have used extensively in the past; 198 ppm iron is similar to the iron content of standard rodent chow and 3 ppm is well below the absolute dietary requirement of ∼50 ppm iron for laboratory rodents. In some mice, facial vein bleeding was performed once/week for 3 consecutive weeks (∼125 µl of blood removed per week), in combination with low-iron diet feeding, to induce more severe iron deficiency.

### Blood and Tissue Collections

Mice were euthanized by CO_2_ narcosis followed by cervical dislocation. Blood was collected by cardiac puncture for hemoglobin and hematocrit determination, and serum ferroxidase (FOX) activity assays. Duodenum with upper jejunum and liver were collected. Light mucosal scrapes of freshly harvested intestinal tissue were collected for RNA isolation, or enterocytes were isolated as previously described [21]. Tissues were otherwise frozen in liquid nitrogen and stored at −80°C for additional analyses.

### Elemental Analyses, Serum Separation, Hemoglobin, and Hematocrit Measurements

Enterocyte iron and copper content was determined by ICP-MS (Michigan State Univ.). Serum and liver copper and iron concentrations were measured in-house by flame atomic absorption spectrometery (AAS), using standard protocols. Blood samples were incubated at 4°C for 4 hrs, and then serum was obtained by centrifugation at 2000 *g* for 10 min. Hemoglobin (Hb) and hematocrit (Hct) levels were measured using a HemoCue Hb analyzer (Hemocue AB) and a Readacrit Hct system (BD Sedi-Cal™), respectively, according to the manufacturers’ instructions.

### Quantitative Reverse Transcriptase-Polymerase Chain Reaction (qRT-PCR)

Total RNA was isolated from intestinal and liver tissue by Trizol reagent, and SYBR-Green qRT-PCR was performed by well-established methods [9], [22]. Primers (listed in Table S1) were designed to span large introns to eliminate amplification from genomic DNA. Each primer pair was independently validated by initially performing standard curve reactions over a series of cDNA dilutions; linear amplification was noted in each case. Furthermore, melt curves routinely showed single amplicons. Mouse cyclophilin mRNA expression was used to normalize expression levels of experimental genes. Mean fold-change of mRNA levels from all experimental groups (i.e. mutant mice in both dietary treatment groups and FeD WT mice) versus the control group (WT mice consuming the control diet) was calculated by the 2^−ΔΔCt^ method [23] and statistical significance was set at *p*<0.05.

### Determination of Iron Absorption and Utilization

Body iron utilization in mice was determined by gavage with ^59^Fe, essentially as described [24]. Briefly, mice were fasted overnight but allowed free access to water *ad libitum* prior to gavage feeding of ^59^Fe-HCl (500 µCi; 1.85 MBq; Perkin Elmer). ∼2.5 µCi ^59^Fe-HCl, diluted into 0.2 mL of a saline solution containing 0.5 M ascorbic acid, 0.15 M NaCl plus 5 µg of FeSO_4_, was administered using an olive-tipped gavage needle under anesthesia (isoflourane). Mice were subsequently fasted for 7 hr and then given free access to chow. ∼24 hours after gavage, mice were euthanized, blood samples were collected and tissues were harvested. The 24 hour time point was chosen as intestinal transit time in mice is ∼11 hr [25], so all unabsorbed ^59^Fe would have been excreted in the feces. Radioactivity in the whole carcass and blood were initially measured by gamma counting (whole body radioactivity), followed by excising tissues and measuring radioactivity in liver and spleen. Percent iron absorption was calculated as 100×(^59^Fe in blood+total carcass/^59^Fe administered by gavage). Iron accumulation was calculated as counts-per-minute (cpm)/ml of blood or cpm/g of tissue.

### Serum Ferroxidase Activity Assay

Serum ferroxidase activity (ferrozine) was determined as previously described [15]. Ferrozine interacts with Fe^2+^, producing a pink-colored complex that absorbs light at ∼ 570 nm. As iron is oxidized, fluorescence intensity thus decreases (i.e. a substrate disappearance assay). 100 µg of total serum protein was mixed with 3 mM ferrozine dissolved in water, and after various time periods, absorbance was read at 570 nm in a µQuant spectrophotometer (Biotek). In all assays, data were corrected for background absorbance of a reference solution (all components without the serum sample), thus controlling for auto-oxidation of ferrous iron. Oxidase activity is presented as changes in A_570_ (Δ A_570_) in comparison to the reference solution. Reciprocal values of data were calculated, so plots are shown as increasing over time, which was intended to increase clarity.

### Statistical Analysis

Results were expressed as mean ± SD when *n*≥3. Statistical analyses were performed by Student’s t test or 2-way ANOVA using GraphPad Prism software.

## Results

### Copper Rescue of Mutant Mice

**Figure 1:**
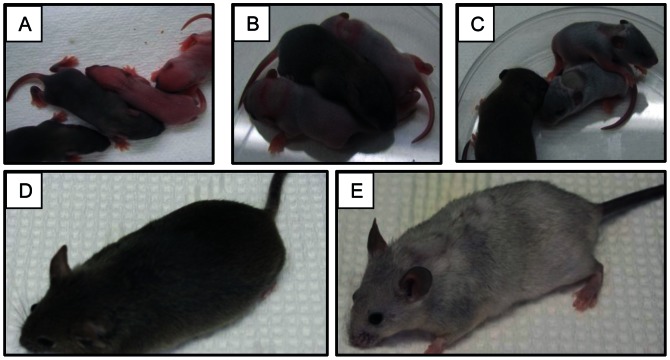
Phenotypic response of Mo*^Br^* ^/*y*^ mice to copper injection. Mice were photographed before and after copper treatment to exemplify the dramatic phenotypical changes that occur in the mutants. (**A**) 7-day-old male mice prior to treatment. Mutant males are pink. (**B**) 8-day-old mice after first Cu injection. (**C**) 9-day-old mice after second Cu injection. (**D**) 3-month-old WT male. (**E**) 3-month-old copper-treated Mo*^Br^* ^/*y*^ male. In panels A, B & C, untreated WT littermates are shown for comparison.

Mo*^Br^* ^/*y*^ mice were born at ratios significantly below predicted Mendelian ratios (based upon the breeding scheme) of 1 Mo^+/y^:1 Mo*^Br^* ^/*y*^. The actual ratio was closer to 3 Mo^+/y^:1 Mo*^Br^* ^/*y*^. This investigation utilized ∼35 total Mo*^Br^* ^/*y*^ mice, which resulted from >250 live births from ∼12 breeding pairs. Except for using some heterozygous females for breeding, WT females and excess WT males were routinely sacrificed in the perinatal period. Copper treatment greatly improved viability of the mutant mice, none of which survived past ∼14 days without treatment. Within 24 hours of injection, strikingly, grey colored hair growth occurred (mutant mice were initially pinkish) (Fig. 1A/B). The changes were even more dramatic after the 2^nd^ copper injection (Fig. 1C). A lesion was noted on the upper back marking the site of injection, which gradually became less distinct and was hardly noticeable 10 weeks later (panel E). The coat color never matched that of the WT mice, but copper-treated Mo*^Br^* ^/*y*^ mice were healthy, showed normal behavior and were similar in size to WT littermates at 3-months-of-age (panels D and E).

### Blood Parameters and Iron Levels in Experimental Mice

**Figure 2:**
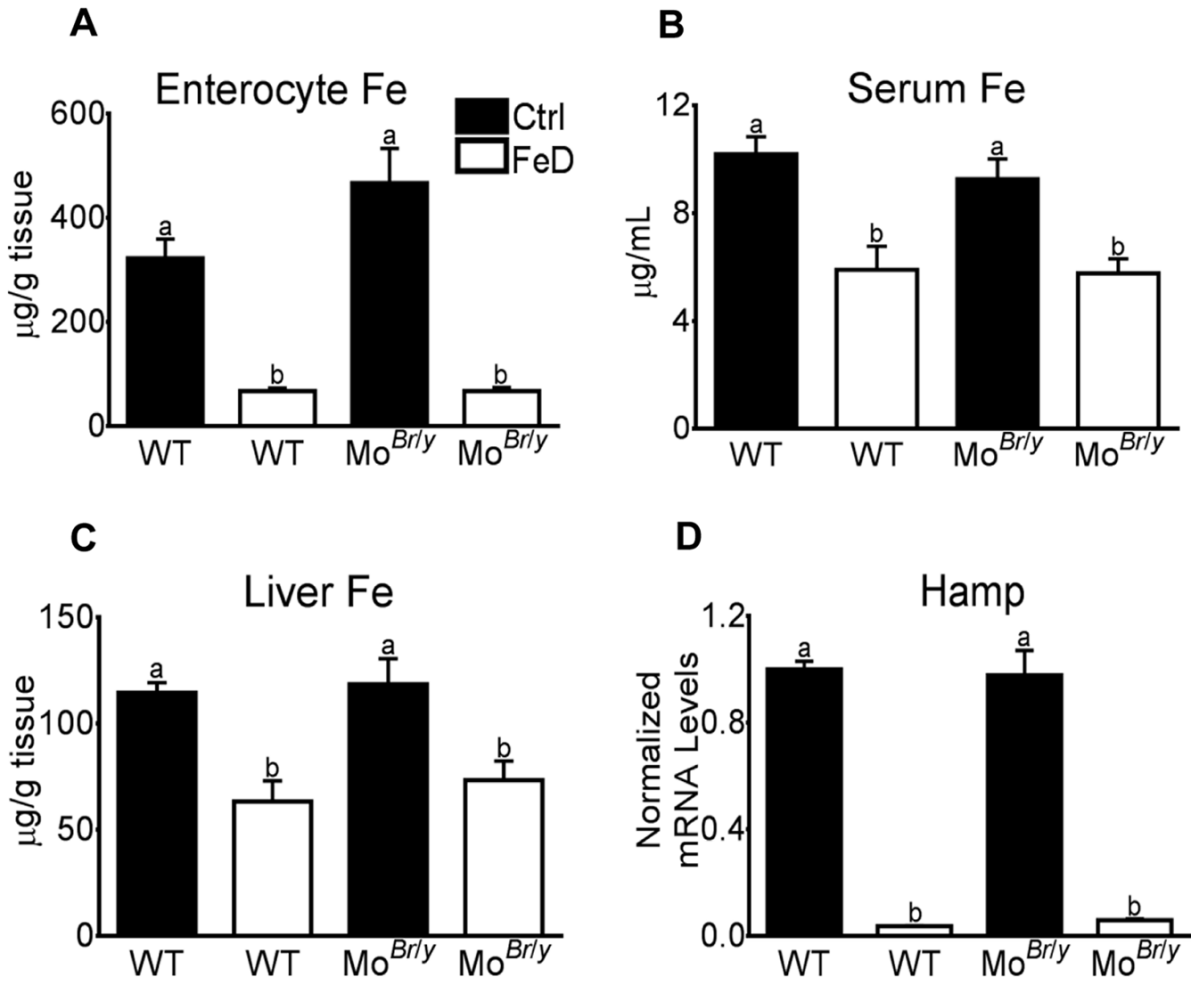
Tissue Fe levels and hepatic hepcidin (Hamp) gene expression. Iron levels were determined as described in *Methods*. Results are depicted graphically, with filled bars representing data from mice fed the control diet and open bars depicting data from FeD mice. Genotype is indicated beneath each bar. Iron levels were normalized by mass of tissue or volume of serum. (**A**) Enterocyte Fe content, n = 3 per group; (**B**) Serum Fe content, n = 4 for WT mice and 3 for Mo*^Br^* ^/*y*^ mice; (**C**) Liver Fe content, n = 10 mice per group. Also depicted is hepatic Hamp mRNA expression (**D**), normalized to cyclophilin mRNA levels (which did not vary significantly). n = 8 per group. *^a,b^* Statistically different from one another within each panel (*p*<0.05). WT, wild-type. Bars (A–D) depict mean±SD.

Mo*^Br^* ^/*y*^ mice were anemic as compared to WT littermates (Hb and Hct decreased by ∼18%; Table 1). Dietary iron deprivation lowered Hb and Hct levels in both genotypes of mice, with the reduction being more dramatic in the Mo*^Br^* ^/*y*^ mice (∼24% from WT levels). Although Mo*^Br^* ^/*y*^ mice were anemic, they had normal enterocyte, serum and liver iron levels, and hepcidin mRNA expression (Fig. 2). Iron-deprived mice of both genotypes however had significant decreases in tissue iron levels and Hamp mRNA expression was dramatically reduced (∼95%; Fig. 2).

**Table 1:** Analysis of blood collected from WT and Brindled mice consuming control (Ctrl) and iron-deficient (FeD) diets.

	Hemoglobin (g/dL)		Hematocrit (%)				
	WT	Mo*^Br^* ^/*y*^		WT	Mo*^Br^* ^/*y*^		
**Ctrl**	15.7±0.71^a^	13.4±0.57^c^		50.9±1.83^a^		46.2±1.91^c^	
**FeD**	14.4±0.86^b^	11.9±0.59^d^		47.6±3.15^b^		41.4±2.88^d^	

Hemoglobin (Hb) and hematocrit (Hct) levels were measured from serum samples taken from experimental mice. *^a,b,c,d^* Statistically different from one another within each panel (*p*<0.05). n = 8 animals per group.

### Quantification of Gene Expression in Mouse Duodenal Mucosa and Liver

**Figure 3:**
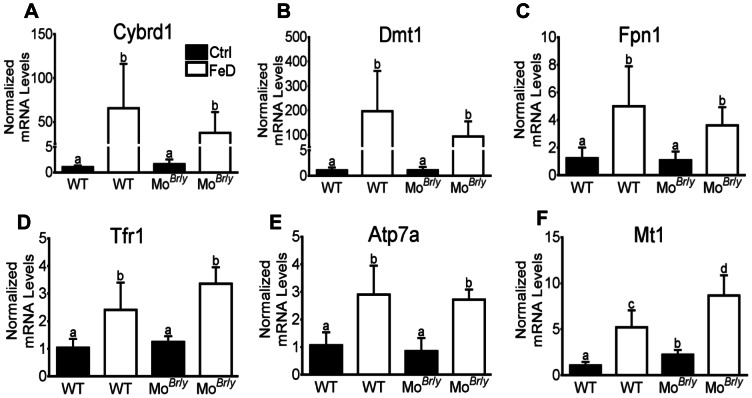
Intestinal qRT-PCR analysis of iron and copper homeostasis-related genes. Expression of key genes (indicated in each panel) was determined by standard methods, with each experimental gene being normalized to expression of mouse cyclophilin mRNA (which did not vary significantly). Filled bars represent data from mice fed the control diet and open bars represent data from mice consuming the low-iron diet. Genotype is indicated below each bar. *^a,b,c,d^* Statistically different from one another within each panel (*p*<0.05). n = 4 for each genotype consuming the control diet and n = 6 for each genotype fed the low-iron diet. WT, wild-type. Bars (A–F) depict mean±SD.

Expression of Cybrd1, Dmt1, Fpn1, Tfr1 and Atp7a increased in both genotypes upon dietary iron deprivation in duodenum samples from experimental animals (Fig. 3). Fold increases however did not achieve statistically significant differences between genotypes. Interestingly, expression of these genes was unchanged in Mo*^Br^* ^/*y*^ mice (as compared to WT littermates), despite the fact that they were anemic. Mt1 expression was increased in Mo*^Br^* ^/*y*^ mice as compared to WT mice (2-fold), and furthermore, induction was noted in both genotypes upon iron deprivation, as compared to control-diet fed mice (Fig. 3). Mt1 induction was greater in the mutant mice (∼1.8×higher). Expression of ferritin (Ftn), Heph and copper transporter 1 (Ctr1) mRNA was not different between genotypes or dietary groups (data not shown).

**Figure 4:**
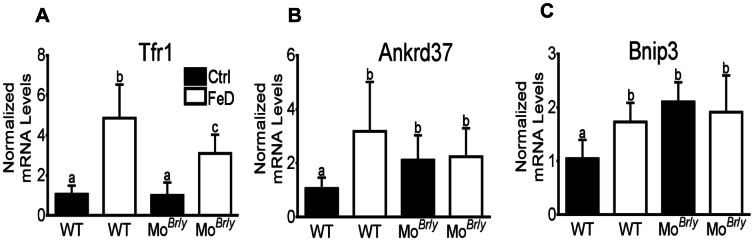
Liver qRT-PCR analysis of iron- and hypoxia-related genes. Expression of key genes (indicated in each panel) was determined by standard methods, with each experimental gene being normalized to expression of mouse cyclophilin mRNA (which did not vary significantly). Filled bars represent data from mice fed the control diet and open bars represent data from mice consuming the low-iron diet. Genotype is indicated below each bar. *^a,b,c,d^* Statistically different from one another within each panel (*p*<0.05). n = 6 for all groups. WT, wild type; Ankrd37, Ankyrin repeat domain 37; Bnip3, BCL2/adenovirus E1B 19 kDa interacting protein 3. Bars (A–C) depict mean±SD.

In the liver, Tfr1 mRNA expression was increased by iron deprivation in both genotypes (∼5 fold in WT mice and 3.5-fold in Mo*^Br^* ^/*y*^ mice) (Fig. 4). Moreover, expression of two known hypoxia-responsive genes, Ankrd37 and Bnip3 were upregulated in all mice that had low circulating hemoglobin levels (i.e. Mo*^Br^* ^/*y*^ mice and both genotypes consuming the low-iron diet). Furthermore, hepatic expression of ceruloplasmin (Cp), Dmt1 and Fpn1 was not significantly different amongst all groups of mice (data not shown).

### Copper Levels in Serum and Tissues of Experimental Mice, and Serum Cp Activity

**Figure 5:**
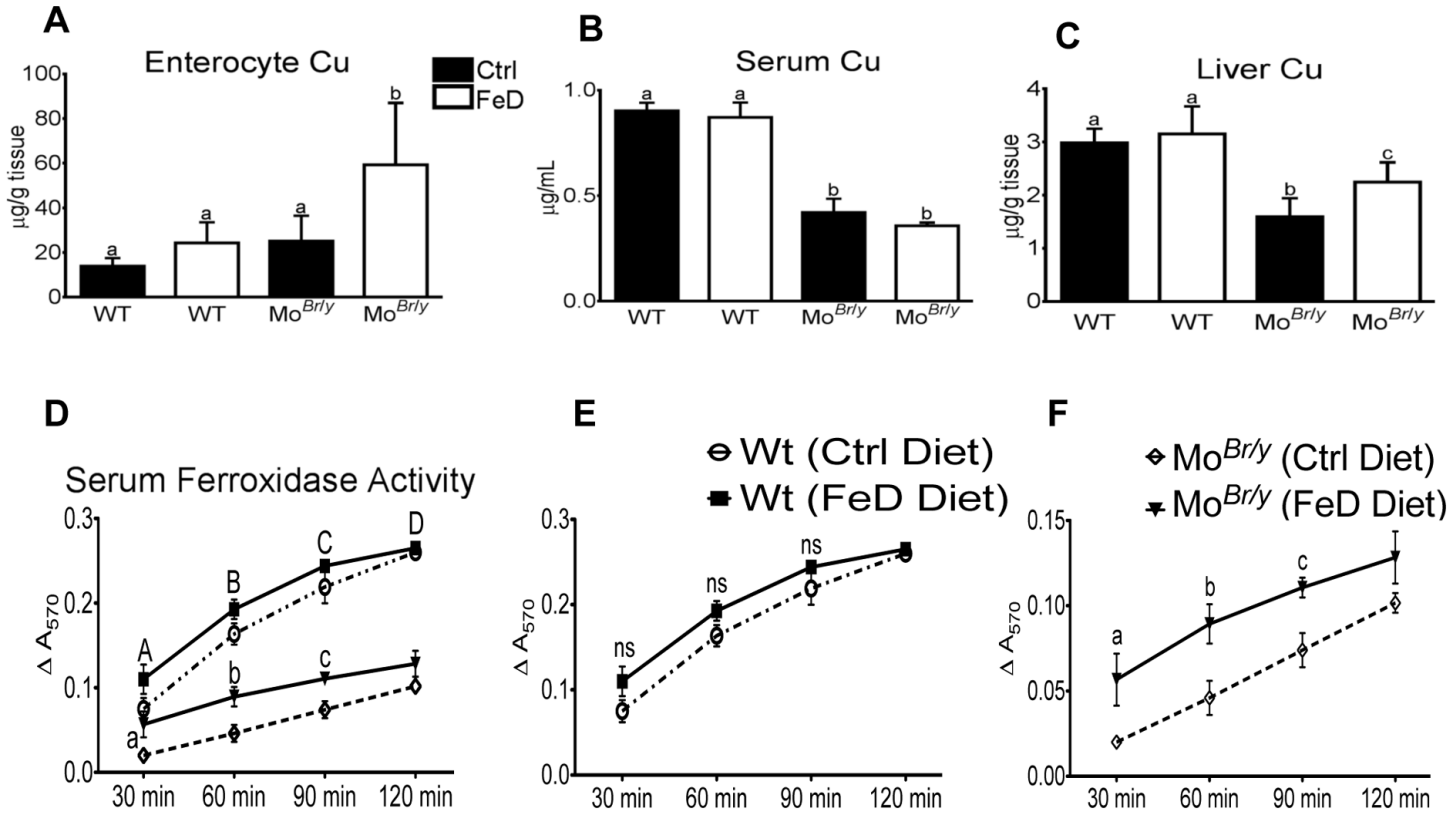
Tissue and serum Cu levels and serum FOX activity in experimental mice. Copper levels were determined as described in *Methods* (**A–C**). Results are depicted graphically, with filled bars representing data from mice fed the control diet and open bars depicting data from FeD mice. Genotype is indicated beneath each bar. Copper levels were normalized by mass of tissue or volume of serum. (**A**) Serum copper levels, n = 4 for both groups of WT mice and n = 3 for both groups of Mo*^Br^* ^/*y*^ mice; (**B**) Enterocyte copper levels, n = 3 for all groups; (**C**) Liver Cu levels, n = 10 for all groups. *^a,b^* Statistically different from one another within each panel (*p*<0.05). Also depicted is quantification of serum FOX activity (**D–F**), as determined by ferrozine assay. Composite data from all 4 groups is shown in panel **D**, while panel **E** shows data from WT mice consuming control or low-iron diets and (**F**) depicts data from Mo*^Br^* ^/*y*^ mice on both diets. (**D**) Uppercase letters indicate that data from both control groups statistically differ from data from both groups of Mo^Br/y^ mice, while lowercase letters indicate significance between the Mo*^Br^* ^/*y*^ mice on the different diets (*p*<0.05 for both); (**E**) ns, not-significant; (**F**) Lowercase letters indicate significance between groups at individual time points (*p*<0.05). (**D–F**) Statistics by 2-way ANOVA followed Bonferroni’s multiple comparisons test. n = 4 for both control groups and n = 3 for both groups of Mo*^Br^* ^/*y*^ mice. Bars (A–F) depict mean±SD.

Enterocyte copper levels were similar in WT mice in both dietary treatment groups and in Mo*^Br^* ^/*y*^ mice consuming the control diet (Fig. 5). In FeD Mo*^Br^* ^/*y*^ mice however, the enterocyte copper content was increased ∼3-fold. Serum copper levels were reduced ∼55% in Mo*^Br^* ^/*y*^ mice consuming both diets, as compared to wild-type mice in both dietary treatment groups. Furthermore, liver copper content, while not differing between WT mice consuming either diet, was reduced ∼46% in Mo*^Br^* ^/*y*^ mice consuming control diet and ∼25% in Mo*^Br^* ^/*y*^ mice consuming the low-iron diet (both as compared to WT mice) (Fig. 5).

Serum FOX activity was not different when comparing the WT mice on either dietary treatment, but was reduced in both groups of Mo*^Br^* ^/*y*^ mice (Fig. 5). FOX activity was however higher in the Mo*^Br^* ^/*y*^ mice consuming the low-iron diet, as compared to those consuming the control diet. For example, at the 60 min time point, serum FOX activity was reduced from control values ∼54% in Mo*^Br^* ^/*y*^ mice but the reduction was only ∼29% in FeD Mo*^Br^* ^/*y*^ mice. At the 90 min time point, in Mo*^Br^* ^/*y*^ mice on control or low-iron diets, the reductions were ∼66% and ∼50%, respectively.

### ^59^Fe Absorption and Utilization After Dietary Iron Deprivation With or Without Concurrent Phlebotomy

**Figure 6:**
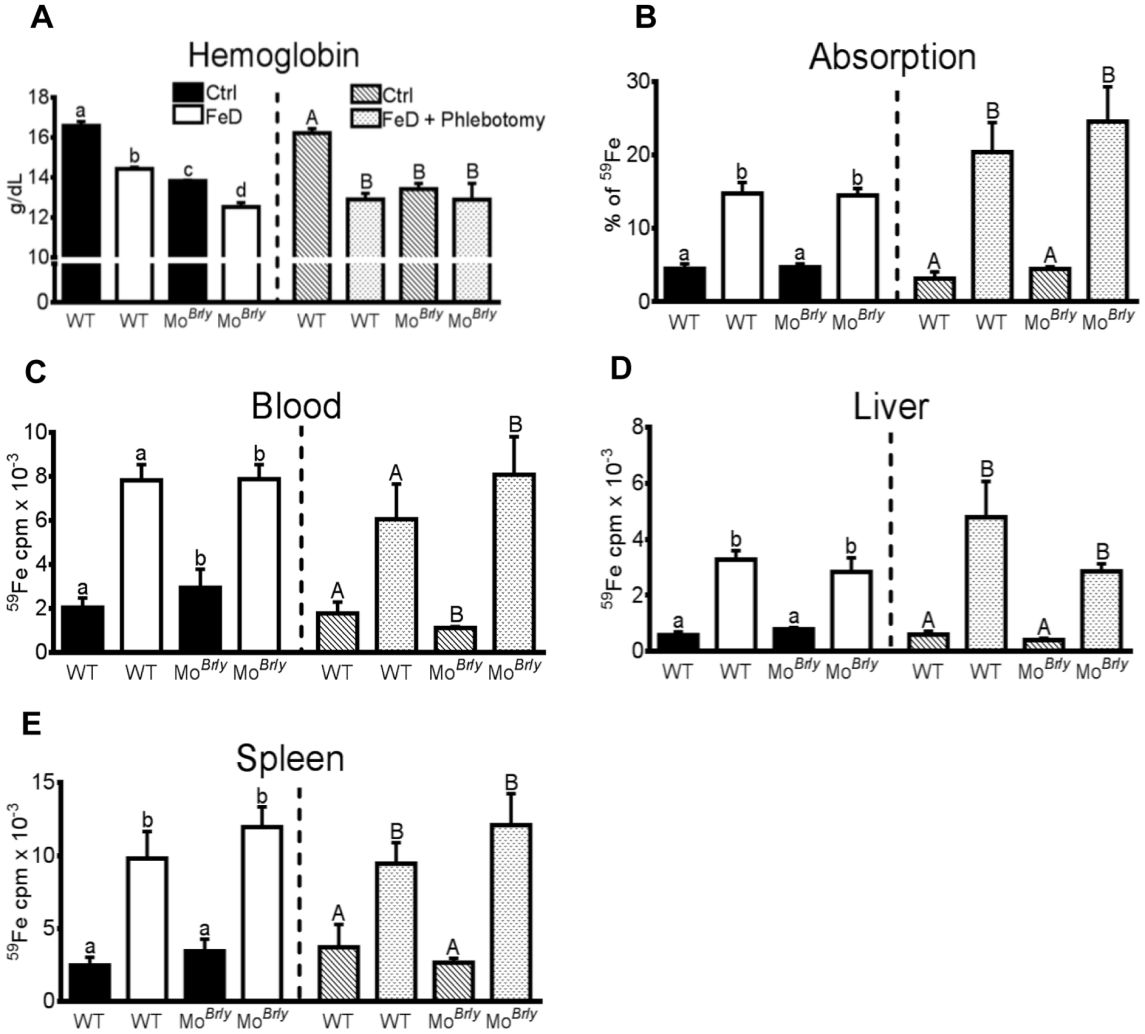
Intestinal iron absorption and whole-body iron utilization in experimental mice deprived of dietary iron with or without concurrent phlebotomy. Mice were fed a control diet or an iron-deficient diet for three weeks with or without concurrent once weekly bloodletting. Subsequently, ^59^Fe was administered by oral gavage and the distribution of radioactivity was assessed 24 hours later. Blood hemoglobin levels are depicted (**A**) as well as % of ^59^Fe dose absorbed (**B**), as an indicator of intestinal iron absorption, and radioactive counts in blood, liver and spleen (**C–E**). In all panels, the left side depicts data from dietary iron deprivation only, while the right panel shows data from dietary iron deprivation plus bleeding. Each bar is defined in panel A, and is consistent throughout all panels. ^a, b^ and ^A, B^ indicate statistical significance between groups within each one half of an individual panel (*p*<0.05). Genotypes are indicated below each bar. For dietary treatment groups, n = 3 for all; for dietary treatment plus phlebotomy groups, n = 5 for WT mice fed the control diet, n = 6 for WT mice consuming the low-iron diet, and n = 4 for Mo*^Br^* ^/*y*^ mice consuming both diets. Bars (A–E) depict mean±SD.

In mice used for iron uptake studies, Hb and Hct levels showed similar reductions to data presented in Table 1 (Fig. 6). Mice that were concurrently bled however displayed more significant Hb and Hct reductions. Iron absorption (% of ^59^Fe dose), and ^59^Fe in blood, liver and spleen was increased to a similar extent in FeD mice of both genotypes, and furthermore, did not vary between WT mice and Mo*^Br^* ^/*y*^ mice consuming the control diet (Fig. 6).

## Discussion

Although copper homeostasis has been thoroughly examined in Brindled mice (as cited above), to our knowledge, iron homeostasis has not been investigated. Suckling mice were previously injected with iron (and compared to copper injection) and killed a few days later for analysis [26], [27]; however, mechanisms of absorption are different in neonatal mice, likely involving non-specific nutrient absorption via pinocytosis and *para*-cellular flux [20]. Direct comparison to the current studies using adult Mo*^Br^* ^/*y*^ mice is thus not meaningful.

Brindled mice die perinatally of severe copper deficiency [19], [28]. Early studies on Mo*^Br^* ^/*y*^ mice demonstrated that they could be rescued by IP injection of copper around the 8^th^ day of life [29]. Copper-treated, mutant mice developed and grew normally but still had persistent perturbations in body copper levels; 53 days post-injection, Cu levels in kidney were high and levels were low in brain and serum [12]. We treated mice twice with copper chloride in the perinatal period, allowed mice to recover for 7–8 weeks and then deprived them of dietary iron. Wild-type littermates did not undergo copper injections, as we predicted it would be without effect since mice were studied ∼11 weeks after treatment of the mutants. Earlier studies treated WT mice with copper, and although liver copper levels were very high 11 days post-injection (7× higher than untreated controls), they had normalized 2 weeks later [12], and no other persistent perturbations in copper levels were noted. Furthermore, although the copper treatment likely produced an acute inflammatory response, when mice were studied at 12-weeks-of-age, there was no sign of inflammation, as exemplified by normal hepatic hepcidin (Hamp) mRNA expression. Hamp is known to be strongly induced at the transcriptional level by pro-inflammatory cytokines (e.g. IL-6) [30], and as such, Hamp gene expression levels serve as a sensitive marker of the acute-phase response.

Adult, Mo*^Br^* ^/*y*^ mice were similar in size to and just as active as WT littermates, had pigmentation defects and were anemic, but had no abnormalities in body iron levels and no changes in Hamp expression. The mutant mice did however have perturbations in body copper levels including decreased hepatic and serum copper, indicative of severe systemic copper deficiency (see Table S2 for a comprehensive overview of all data from this study). Low hepatic copper levels have been noted in Brindled mice [31], although this is thought to be a secondary phenomenon related to copper being avidly taken up by and trapped within other tissues [19]. Serum FOX (i.e. Cp) activity was also reduced in the rescued mutants, consistent with previous observations. In the current investigation, a significant relationship between serum and liver copper and serum FOX activity was documented, when considering all groups of experimental mice together (r = 0.8852 for serum copper vs. serum FOX activity, and r = 0.9552 for liver copper vs. serum FOX activity) (Fig. S1). This is consistent with our previous observations in rats consuming various iron- and copper-deficient diets [15]. Given the documented role of Cp in iron release from liver and other tissues [32], it is not readily apparent why liver iron levels were not increased in the mutant mice. In sum, these observations demonstrated that copper-treated, Brindled mice suffered from copper-deficiency anemia, displaying many known symptoms of copper deprivation in mice [33]. Interestingly, this is in contrast to phenotypical changes associated with copper deficiency in other mammalian species, including swine, rats and humans [34]–[36], in which perturbations in iron homeostasis are noted. These differences could be species-related or due to the underlying genetic defect in Mo*^Br^* ^/*y*^ mice.

The main goal of this investigation was to define the role of Atp7a and copper in the compensatory response of the intestinal epithelium to iron deprivation. Accordingly, iron absorption studies were performed in all experimental mice, including WT, iron-deficient (FeD) WT, Brindled and FeD Brindled mice. FeD WT mice had the classical iron-deficient phenotype. Although body copper levels were not altered in the FeD WT mice, as is commonly noted in other mammalian species (e.g. human, rat etc.), metallothionein and Atp7a gene expression increased in duodenal enterocytes perhaps indicating subtle alterations in copper homeostasis. As expected, iron absorption in WT FeD mice was significantly enhanced by iron deprivation and was further increased by concurrent phlebotomy.

Data interpretation from the Mo*^Br^* ^/*y*^ mice was more complex given the underlying copper deficiency associated with both dietary treatment groups. The mutants consuming a standard chow diet were copper deficient, but had no observable perturbations in iron homeostasis, in contrast to previous studies on dietary copper deficiency in mice [37], [38] and rats [39], in which hypoferremia was noted. It was hypothesized that Heph expression/activity was decreased by copper deprivation and that this reduced iron release from enterocytes causing hypoferremia, consistent with the phenotype of *Heph* mutant (sex-linked anemia [*sla*]) mice [40]. In the case of the Mo*^Br^* ^/*y*^ mice, although they had systemic copper deficiency, enterocytes were not depleted of copper (probably due at least in part to the decreased copper export function of Atp7a), perhaps explaining why iron absorption was not affected. This could also explain why the Mo*^Br^* ^/*y*^ mice did not have low serum iron and were not iron deficient, although such a conclusion would require experimental validation. Past studies have provided conflicting results in regards to iron homeostasis in the setting of dietary copper deprivation in mice [41]–[43].

Despite the anemia and probable intestinal hypoxia in Mo*^Br^* ^/*y*^ mice, documented Hif2α targets in duodenal enterocytes (e.g. Dmt1, Fpn1, Atp7a) were not induced. Known hypoxia-responsive genes were indeed induced in liver of the mutant mice (Ankrd37 [44], Bnip3 [45]), consistent with decreased blood hemoglobin levels (and tissue hypoxia). However, when Mo*^Br^* ^/*y*^ mice were deprived of dietary iron, Hif2α target genes were induced (as in WT FeD mice), consistent with previous observations [46]–[48]. These observations suggest that the signal for Hif2α-mediated induction of iron and copper homeostasis-related genes in mice is low intracellular iron, as hypoxia in the setting of normal intracellular iron levels did not trigger the response. This possibility would however have to be experimentally verified before definitive conclusions could be drawn.

Of further note is the fact that although circulating FOX (i.e. ceruloplasmin) levels increased during FeD of Mo*^Br^* ^/*y*^ mice, no changes in hepatic *Cp* mRNA expression were observed. This is in contrast to the reported Hif1α-mediated transcriptional induction of the Cp promoter in HepG2 cells [49], but is consistent with our previous study in rats in which iron deprivation increased hepatic Cp protein expression and serum Cp activity without effect on Cp mRNA expression [15]. Thus, in vivo upregulation of Cp in rats and mice during iron-deficiency anemia (and hypoxia) likely does not involve HIF signaling in the liver.

In summary, the compensatory response of the WT mice used in this study (CBA/C3H) to iron deprivation does not involve concurrent alterations in enterocyte or liver copper levels or changes in serum FOX activity. This exemplifies a perhaps unique aspect of iron homeostasis in this strain of mice, as altered copper homeostasis typifies iron deficiency in humans and other mammalian species. Conversely, in the same strain of mice harboring a 6 bp deletion in the *Atp7a* gene, enhanced iron absorption upon iron deprivation was associated with increased enterocyte and hepatic copper levels and serum FOX activity. The well-established interrelationship between iron and copper is thus preserved in the Mo*^Br^* ^/*y*^ mice. These findings raise two important questions: 1) are alterations in copper homeostasis necessary for the Mo*^Br^* ^/*y*^ mice to appropriately upregulate intestinal iron absorption, and 2) how can hepatic copper levels and serum FOX activity increase in the absence of an essential intestinal copper exporter? In regards to question 1, we speculate that a threshold of enterocyte and hepatic copper levels is required to maintain iron homeostasis and given that Mo*^Br^* ^/*y*^ mice were copper deficient, increases in copper levels during iron deprivation were necessary to achieve that threshold (and support iron homeostasis). Question 2 has two possible explanations, first that residual Atp7a function could explain increased hepatic copper levels, or secondly that hepatic copper loading is independent of copper absorption and relates to altered copper excretion, as mediated by the Atp7b copper-transporting ATPase expressed on the canicular surface of hepatocytes. Although definitive answers to these puzzling questions await further experimentation, these novel studies provide additional support of the concept that copper plays an important role in the maintenance of mammalian iron homeostasis.

## Supporting Information

Figure S1 **Relative Cp activity as a function of serum and liver copper levels.** Plots show the relationship between serum (A) and liver (B) copper and Cp activity. Lines fitting the data were derived by linear regression for Cp activity versus serum and liver copper. In panels A and B, *P*<0.0001. r, Pearson correlation coefficient.
(TIF)

Table S1 **Sequences of Oligonucleotide Primers Used for qRT-PCR Analysis.** Forward (F) and reverse (R) primers used for PCR analysis of gene expression are listed. Cyclophilin was utilized as an internal standard for normalization of experimental gene expression.
(TIF)

Table S2 **Summary of Iron- and Copper-Related Parameters in Experimental Mice.** A summary of all data obtained in this investigation is listed. Parameters that were not different between groups are shaded blue. Yellow shading indicates an increase in this parameter as compared to WT mice. Fold increases were estimated by averaging the values from increased groups that were not statistically different from one another. Green shading indicates parameters that were decreased as compared to WT mice. Percent of the WT values are shown. Percent of WT values were estimated by averaging the values from decreased groups that were not statistically different from one another.
(TIF)
