# Supplementary figures and images for: Investigation of Iron Metabolism in Mice Expressing a Mutant Menke’s Copper Transporting ATPase (Atp7a) Protein with Diminished Activity (Brindled; MoBr /y)
Source: PLoS One. 2013 Jun 11;8(6):e66010. doi: 10.1371/journal.pone.0066010 (PMC3679098; doi:10.1371/journal.pone.0066010)

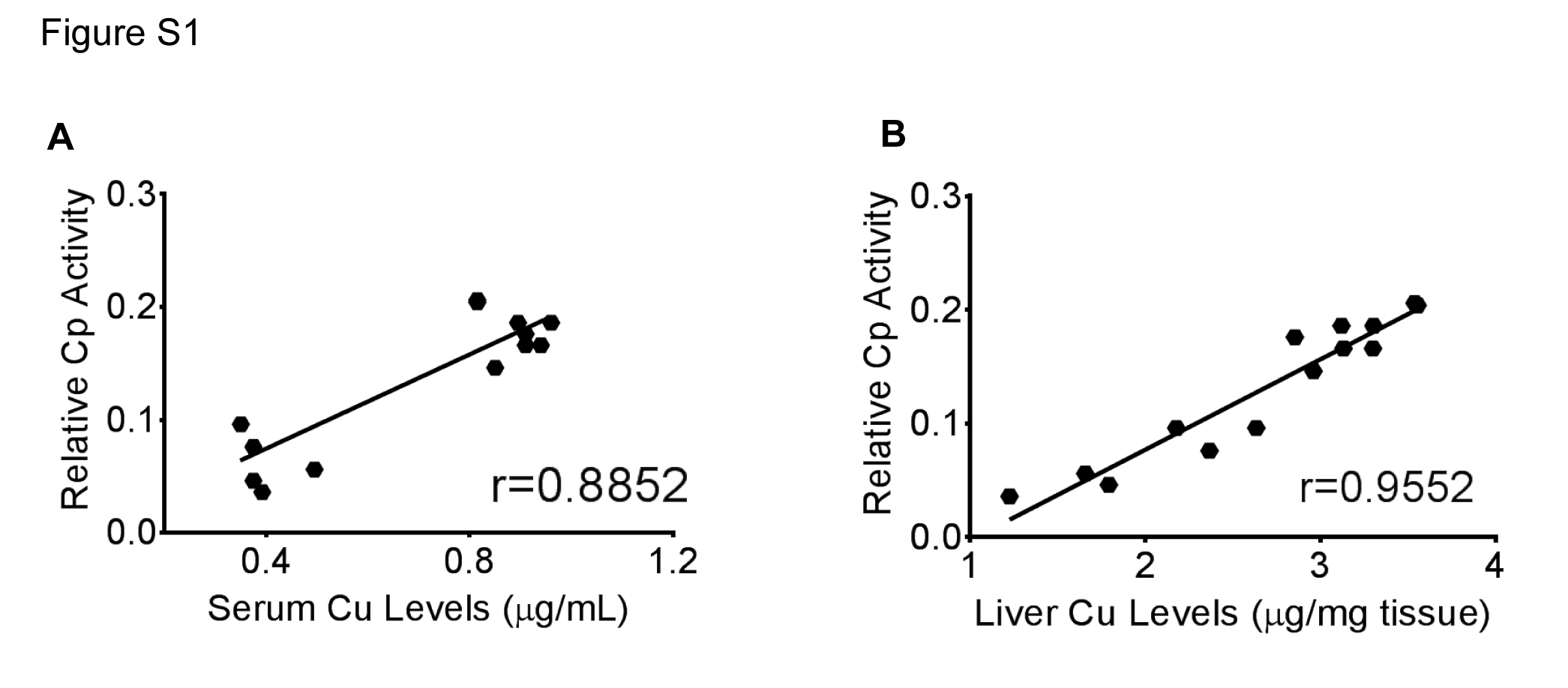

Supplement: Figure S1 — Relative Cp activity as a function of serum and liver copper levels. Plots show the relationship between serum (A) and liver (B) copper and Cp activity. Lines fitting the data were derived by linear regression for Cp activity versus serum and liver copper. In panels A and B, P<0.0001. r, Pearson correlation coefficient. (TIF) [file pone.0066010.s001.tif]

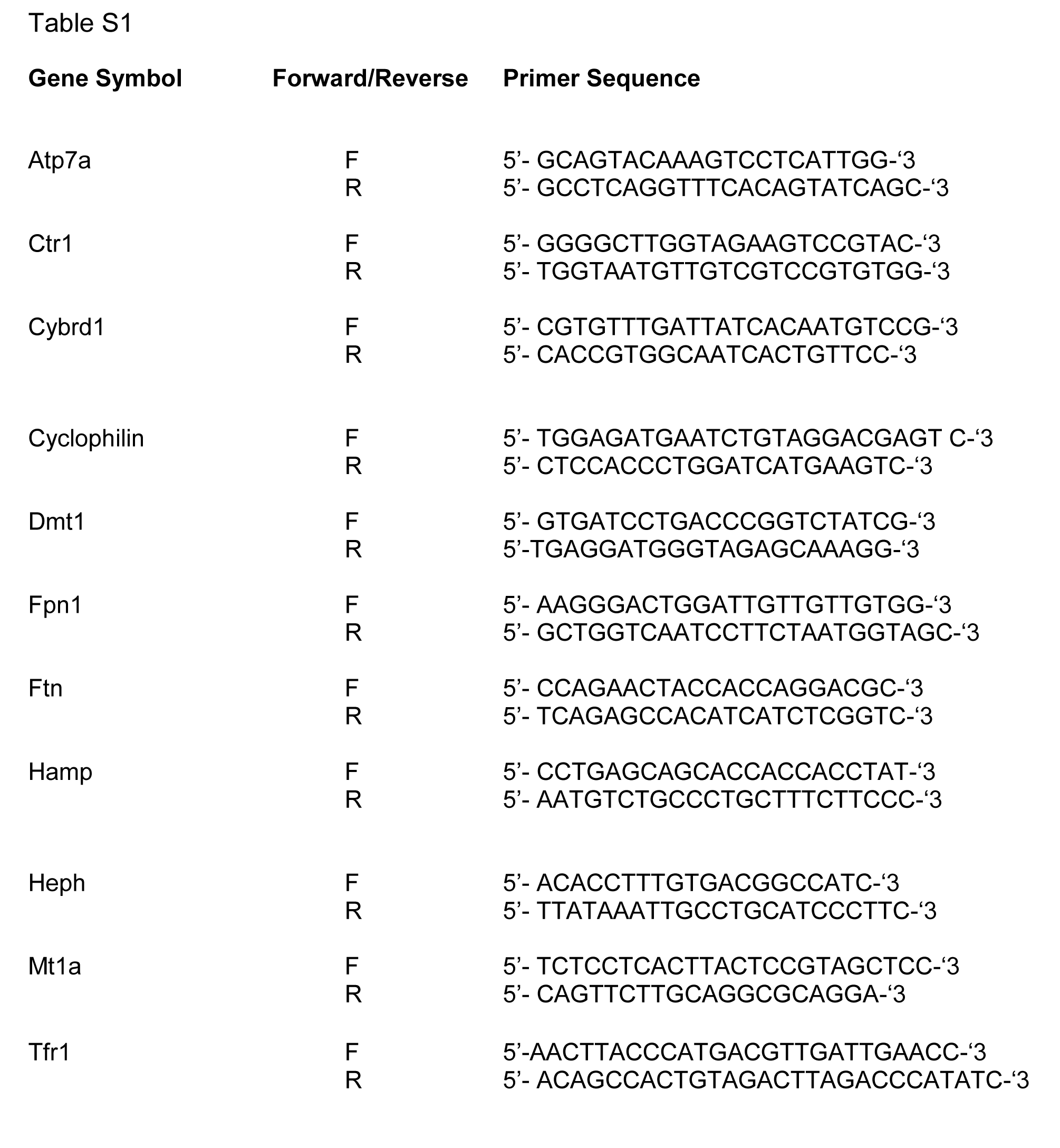

Supplement: Table S1 — Sequences of Oligonucleotide Primers Used for qRT-PCR Analysis. Forward (F) and reverse (R) primers used for PCR analysis of gene expression are listed. Cyclophilin was utilized as an internal standard for normalization of experimental gene expression. (TIF) [file pone.0066010.s002.tif]

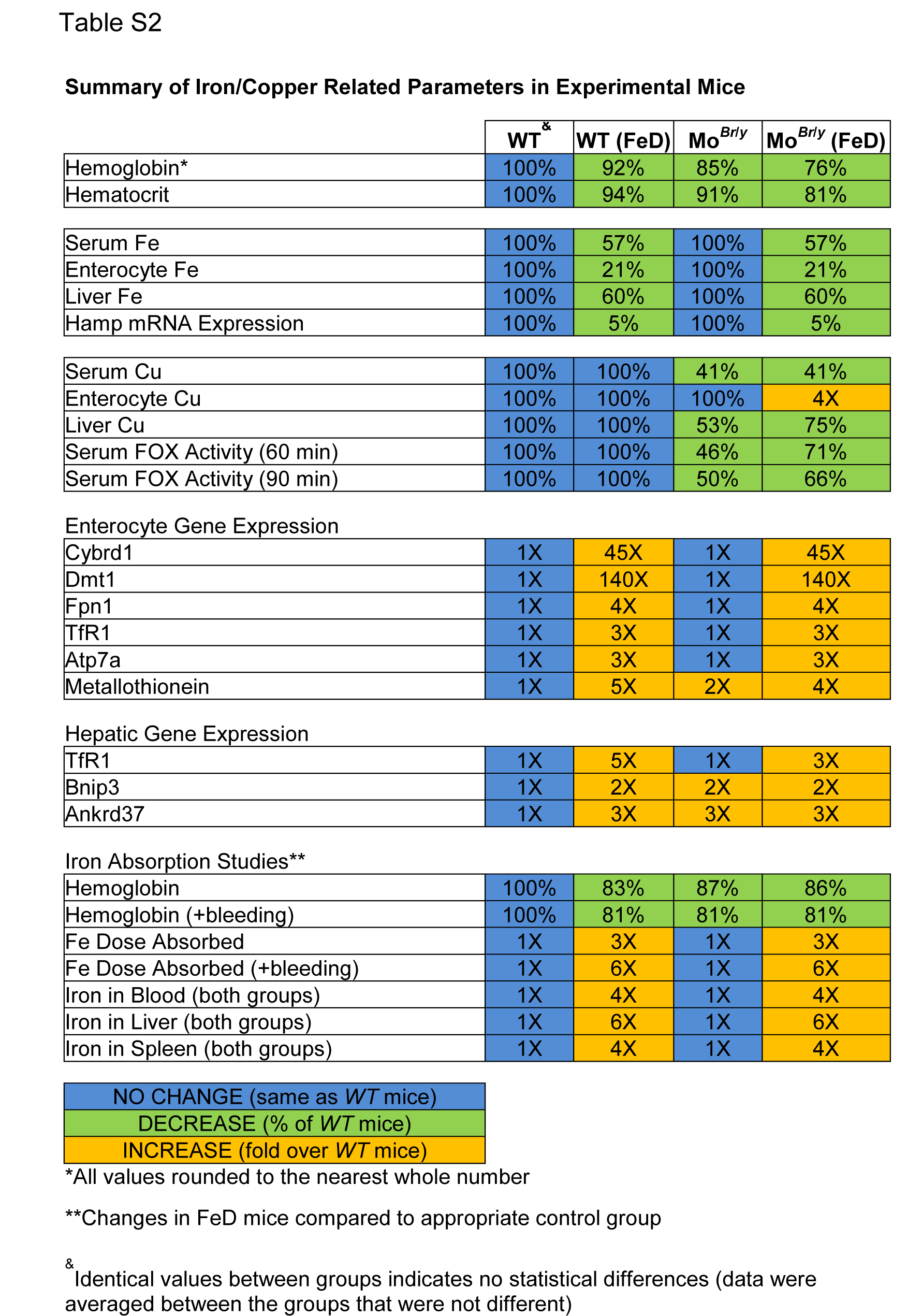

Supplement: Table S2 — Summary of Iron- and Copper-Related Parameters in Experimental Mice. A summary of all data obtained in this investigation is listed. Parameters that were not different between groups are shaded blue. Yellow shading indicates an increase in this parameter as compared to WT mice. Fold increases were estimated by averaging the values from increased groups that were not statistically different from one another. Green shading indicates parameters that were decreased as compared to WT mice. Percent of the WT values are shown. Percent of WT values were estimated by averaging the values from decreased groups that were not statistically different from one another. (TIF) [file pone.0066010.s003.tif]
